# Type B insulin resistance syndrome with Scleroderma successfully treated with multiple immune suppressants after eradication of Helicobacter pylori infection: a case report

**DOI:** 10.1186/s12902-016-0099-5

**Published:** 2016-05-03

**Authors:** Guo-Qing Yang, Yi-Jun Li, Jing-Tao Dou, Bao-An Wang, Ju-Ming Lu, Yi-Ming Mu

**Affiliations:** Department of Endocrinology, Chinese PLA General Hospital, No. 28th Fu Xing Road, Beijing, 100853 China

**Keywords:** Type B insulin resistance, Systemic scleroderma, Helicobacter pylori infection

## Abstract

**Background:**

Type B insulin resistance is a rare autoimmune disease characterized by the presence of autoantibodies against the insulin receptor. Helicobacter pylori (H pylori) infection may play a causative role in the autoimmune diseases.

**Case presentation:**

Here, we present a rare case of a 48-year old female patient, who had type B insulin resistance with systemic scleroderma and was successfully treated with multiple immune suppressants after eradication of Helicobacter pylori infection.

**Conclusion:**

The present case suggests H pylori infection-related pathological mechanism may contribute to type B insulin resistance syndrome and autoimmune disorders. Treatment toward H pylori may be helpful to relieve syndrome of type B insulin resistance for H pylori positive patients.

## Background

Type B insulin resistance is a rare autoimmune disease characterized by the presence of anti-insulin receptor antibodies, which impair the binding of insulin to its receptor and cause severe insulin resistance (IR) [1, 2]. The majority of patients present with severe hyperglycemia and extreme insulin resistance because of the insulin receptor-antagonizing action of the autoantibodies, but some patients may present with hypoglycemia due to agonist activity of anti-insulin receptor antibodies [3]. Type B insulin resistance often occurs to patients with autoimmune diseases, such as systemic sclerosis, systemic lupus erythematosus and Sjogren’s syndrome [1]. The exact etiology of these autoimmune diseases remains unclear, but it has long been suggested that exposure to certain environmental agents, such as viral and bacterial infection, in genetically susceptible individuals may be the catalyst for the initiation of autoimmune processes [4, 5]. *Helicobacter pylori* (*H pylori*) infection can affect the host immune response by a number of means and may play a causative role in the autoimmune diseases [4]. Thus, therapy focusing on the elimination of *H pylori* may be helpful to induce the remission of these autoimmune diseases. In this report, we describe the clinical course of a female patient with type B insulin resistance and scleroderma, who was successfully treated with multiple immunosuppressants after eradication of *H pylori* infection.

## Case presentation

A 48-year-old Chinese woman presented with polydipsia and polyuria in January 2011. Her fasting blood glucose (FBG) and postprandial blood glucose (PBG) were 5.41 mmol/L and 25.79 mmol/L respectively and HbA1c was 11.9 % (normal range 4.1–6.5 %). On physical examination, her weight was 43 kg, height 149 cm, BMI 19.4 kg/m^2^. She had no family history of diabetes. The patient was initially diagnosed as type 2 diabetes followed treatment with metformin, pioglitazone and acarbose. The treatment did not relieve her symptoms and her blood glucose could not be controlled.

The patient also complained of Raynaud’s phenomenon and morning stiffness in the joints of her both hands in the past 2 years. Skin hardening was observed in her hands, and her fingers were swollen and hard to flex. The first admission laboratory tests were shown in Table 1. Her liver function and kidney function were normal. Erythrocyte sedimentation rate (ESR), C-reactive protein (CRP) and rheumatoid factor were in normal range. γ-globulin was 30.4 %(11.1–18.8 %. Serological data showed increased levels of IgG and Ig E, decreased levels of serum C3, and positive antinuclear antibodies (1:1000). Anti-glutamic acid decarboxylase (GAD) and anti-insulin antibodies were negative, but anti-insulin receptor antibodies were detected (ELISA, Abcam, China) (strong positive). The response to a 75-g oral glucose tolerance test (75 g-OGTT) was clearly abnormal (Table 2) with marked hyperglycemia and hyperinsulinemia. Leptin and adiponectin levels were 16 pg/mL(102 ± 8 9 pg/mL) and 35.8 ng/mL (5.6 ± 3.2 ng/mL) respectively. Microcirculation nailfold test showed the decreased number of microvessels and irregularly enlarged loops and capillary hemorrhage in her both hands. According to the above manifestations, the patient was diagnosed with type B insulin resistance with Scleroderma.

**Table 1:** Biochemical and Serological Evaluation

Item	1st admission	2nd admission	3rd admission	Reference range (Unit)
Platelets	110	154	130	10^9^/L
Creatinine	36.3	33.8	39.9	44–106 μmol/l
Total protein	67.0	65.7	66.2	55–80 g/L
Albumin	30.7	36.4	39	35–50 g/L
ALT	23.4	29.5	15.5	0–35 U/L
AST	19.1	19.7	19.6	0–35 U/L
Cholesterol	2.88	2.88	2.79	3.1–5.7 mmol/L
Triglyceride	0.56	0.56	0.48	0.4–1.7 mmol/L
LDL-C	0.88	1.79	1.05	mmol/L
HDL-C	1.46	1.02	1.59	mmol/L
IGF-1	25	---	---	116–358 ng
TT_4_	95.6	69.6	66.0	55.3–160.88 nmol/L
FT_4_	9.63	10.4	10.66	10.42–24.62 pmol/L
TT_3_	0.79	0.68	1.03	1.01–2.95 nmol/L
FT_3_	2.46	2.19	3.33	2.76–6.3 pmol/L
TSH	0.80	0.55	0.66	0.35–5.5 mU/L
TgAb	95.2	32.8	10.5	<60 IU/L
TPOAb	105.2	101.1	21.8	<60 IU/L
UFC	198.7	---	--	153.2–789.4 mmol/24 h
FSH	3.91	2.02		1.5–33.4 IU/L
LH	0.55	0.15		0.5–76.3mIU/ml
Testosterone	1.22	1.07		0.5–2.6 nmol/L
ICA	negative	--	--	Negative
IAA	negative	---	--	Negative
GAD	negative	---	--	Negative
INSR antibody	Positive (+++)		Negative	Negative
DOB(Urea breath test)	--	19.7	0	≤4.0
IgE	1630	887	523	0–100 IU/ml
IgG	1960	1720	1650	700–1600 mg/ml
C3	29.8	56.5	73.3	90–180 mg/dl
C4	12.9	13.4	14.9	10–40 mg/dl
ANA	1:1000	>1:1000	1:640	Negative

*DOB* delta over baseline, *TPO-Ab* antithyroid peroxydase antibody, *Tg-Ab* antithyroglobulin antibody, *ANA* Antinuclear antibodies

**Table 2:** Pre- and post-treatment serum levels of blood glucose, insulin and C-peptide after 75 g oral glucose tolerance test

	0 min		60 min		120 min	
	Pre-treatment	Post treatment	Pre-treatment	Post treatment	Pre-treatment	Post treatment
Blood glucose (mmol/L)	4.47	6.45	13.56	7.46	17.51	8.56
Seruminsulin (mU/L)	118.7	10.57	192.1	145.5	452.7	235
C-peptide(ng/mL)	1.33	0.87	2.65	1.79	6.78	3.69

Anti-hyperglycemic agents were initiated, but there was minimal response to multiple oral medications given at high doses, including glymepiride 6 mg/day, metformin 2.0 g/day, and pioglitazone 30 mg/day. Insulin therapy was added, but her blood glucose was higher than 15 mmol/L in the daytime despite that doses were up to 18,000 units/day with continuous insulin (including human regular insulin and rapid-acting insulin) infusion. Methylprednisolone (600 mg/day) was infused for 3 days, followed by prednisone (30 mg/d) and azathioprine (100 mg/d). After 4 weeks, prednisone dose was gradually reduced by 5 mg weekly until a 5-mg maintenance dose was reached, followed by an additional 8 weeks in maintenance dose. These drugs were withdrawn after 16 weeks. Glymepiride, metformin, and pioglitazone were continued, and subcutaneous insulin injections were prescribed (regular human insulin 20 U before every meals, and NPH 10U at bedtime).

**Fig. 1:**
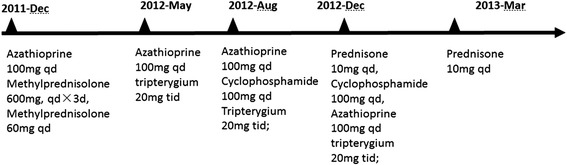
Various immunosuppressive therapies for the patient over time

In her second hospitalization in May 2012, her HbA1c was elevated at 15.3 %. She complained stomachache, and *H pylori* infection was diagnosed based on the carbon-13 urea breath test. She was treated with quadruple eradication therapy (amoxicillin, esomeprazole, clarithromycin and bismuth potassium citrate) for 2 weeks. Her immunosuppressive therapy switched to tripterygium 20 mg tid,azathioprine (100 mg/d) and cyclophosphamide 60 mg/d (Fig. 1). Thereafter, the skin lesion of hands and Raynaud’s phenomenon improved gradually. Meantime, her glycemic control began to improve. HbA1c was decreased to 10.6 and 8.7 % on August and December 2012 respectively, without hypoglycemic episodes. Carbon-13 urea breath test was repeated in Dec 2012 and the result was negative. In the beginning of 2013, she experienced hypoglycemic symptoms, with blood glucose levels between 3.4 to 4.6 mmol/L.

In March 2013, on her third admission, all biochemical and serological parameters were repeated (Table 1). Her HbA1c levels were normal at 5.9 % though 75-g OGTT (Table 2) showed that the hyperglycemia and hyperinsulinemia were improved significantly. Repeated insulin receptor antibody testing revealed that her insulin receptor antibody had become negative. Serological data showed that Ig G, Ig E and complement levels were near normal, and titer of antinuclear antibodies was decreased. Urea breath test of *H pylori* remained negative. At further follow-up, the patient remained euglycemic and no any anti-hyperglycemia therapy was prescribed.

## Conclusions

The case presents a patient who had extremely high endogenous insulin secretion but remained hyperglycemic (glucose levels 16.7–27.8 mmol/l) even on intravenous insulin (either regular or aspart) doses as high as 18,000 U/day, thus she had severe insulin resistance. There are two syndromes associated with severe insulin resistance, type A and type B. Type A insulin resistance syndrome is a genetic disease resulting from mutations in the insulin receptor gene. Type B insulin resistance is caused by autoantibody directed against the insulin receptor. The diagnosis of type B insulin resistance syndrome is based on the presence of hyperglycemia (sometimes hypoglycemia paradoxically), hyperinsulinemia and positive insulin receptor antibodies. Our patient presented with severe hyperglycemia and hyperinsulinemia, and her other clinical features were also consistent with type B insulin resistance described by Arioglu E et al. [1]. Besides, the diagnosis of type B insulin resistance was confirmed by the presence of insulin receptor antibody.

Type B insulin resistance syndrome is frequently associated with other autoimmune diseases. Based on literature review, there have been only one case report of type B insulin resistance associated with scleroderma [6]. Scleroderma (Systemic sclerosis) is a heterogeneous disease typically presented with 3 hallmarks: small vessel vasculopathy, production of autoantibodies, and fibroblast dysfunction leading to increased deposition of extracellular matrix [7]. Typical scleroderma is classically defined as symmetrical skin thickening, with Raynaud’s phenomenon presented in about 90 % of cases, nail-fold capillary changes, and antinuclear antibodies [7]. Systemic involvement may or may not be present. Additional symptoms of scleroderma typically present with two years’ Raynaud’s phenomenon. In our case, the patient had typical skin changes, nail-fold capillary abnormalities, antinuclear antibodies detected by biochemical and serologic tests, elevated Ig E and Ig G, and low level of complement (C3). These manifestos collectively led to the diagnosis of scleroderma according to ACR/EULAR (American College of Rheumatology/European League Against Rheumatism) classification criteria of systemic sclerosis [8]. So it was confirmed that type B insulin resistance and scleroderma occurred concomitantly. However, the mechanisms for the development of anti-insulin-receptor antibodies in this autoimmune disorder remain unknown.

*H pylori* may play a pathogenetic role in the development of these two conditions. *H pylori* is a spiral-shaped, flagellated, Gram-negative bacterium, which is directly implicated in several gastrointestinal diseases such as dyspepsia, acute and chronic gastritis, and peptic ulceration [9]. *H pylori* is associated with some extra-gastric diseases, including autoimmune diseases and endocrine diseases. Some reports suggested that HOMA -IR in *H pylori* positive patients was higher than that in *H pylori* negative ones [10]. But there is only one case available, which suggests *H pylori* infection could be underlying pathological mechanism of type B insulin resistance syndrome. Meanwhile, some studies suggest a possible role of *H pylori* in the development of scleroderma [11–15]. In a much larger cohort of 124 Japanese patents with systemic sclerosis, prevalence of anti- *H pylori* antibodies was reported as 55.6 %—much higher than that in the healthy controls [16]. Regarding to the mechanism, It is hypothesized that *H pylori* induces immune dysregulation and aggravating the course of scleroderma [17].

The treatment of type B insulin resistance should focus on the improvement of insulin resistance, glucose abnormalities, and the immune disorders. The anti-hyperglycemia therapy includes insulin therapy and oral hypoglycemic agents. The dose of insulin needed to control hyperglycemia is typically high. Insulin sensitizers are usually prescribed in this condition, but the efficacy of these drugs has not been clearly studied. Nevertheless, metformin has been reported to improve insulin resistance and reduce the dose of exogenous insulin needed to control hyperglycemia in patient with anti-insulin receptor antibodies [18]. Various immune-modulating therapies such as corticosteroids, plasmapheresis, cyclophosphamide, cyclosporine, azathioprine, mycophenolate mofetil, and rituximab have been tried. Corticosteroids are the most commonly used medicine to control the autoimmune process. In addition, cyclophosphamide or plasmapheresis has been successfully used in type B insulin resistance syndrome. The patient had no response to the combined therapy including insulin and several oral hypoglycemic agents. Initially, a combination therapy including steroids, azathioprine and tripterygium failed to reduce the disease activity in this case. However, the patient’s blood glucose improved gradually and skin lesions relieved after therapies aiming at *H pylori* infection on the basis of combined immunotherapy. Until now, it is unclear whether *H pylori* eradication improves skin manifestations of scleroderma and insulin resistance in patients. At least, in primary Raynaud’s phenomenon, eradication of *H pylori* infection was reported to be associated with complete remission in some cases and relieved symptoms in most of the treated patients [19]. *H pylori* eradication also ameliorated type B insulin resistance syndrome [20].

The present case suggests *H pylori* infection-related pathological mechanism may contribute to type B insulin resistance syndrome and autoimmune disorders. In cases of type B insulin resistance syndrome, testing for *H pylori* infection may be worthwhile and eradication of *H pylori* should be considered as a therapeutic option, which may relieve the syndrome of type B insulin resistance.

### Declarations

#### Ethics and consent to participate statement

This study was approved by the Ethics Committee of Chinese People’s Liberation Army General Hospital, China. Written informed consent was obtained from the patient for publication of this Case report. A copy of the written consent is available for review by the Editor of this journal.

### Consent to publish statements

Consent for publication of the data was obtained from the patient.

### Availability of data and materials statement

All the data supporting the findings in this study are presented in this article.

## Abbreviations

H pylori: Helicobacter pylori
IR: insulin resistance
FBG: fasting blood glucose
PBG: postprandial blood glucose
ESR: Erythrocyte sedimentation rate
CRP: C-reactive protein
GAD: glutamic acid decarboxylase
